# Antimicrobial Activity of Amino-Modified Cellulose Nanofibrils Decorated with Silver Nanoparticles

**DOI:** 10.3390/jfb15100304

**Published:** 2024-10-13

**Authors:** Vesna Lazić, Jovan M. Nedeljković, Vanja Kokol

**Affiliations:** 1 Vinča Institute of Nuclear Sciences—National Institute of the Republic of Serbia, University of Belgrade, Centre of Excellence for Photoconversion, 11000 Belgrade, Serbia; vesna.lazic@vin.bg.ac.rs; 2 Faculty of Mechanical Engineering, University of Maribor, 2000 Maribor, Slovenia

**Keywords:** amino-modified cellulose nanofibrils, silver nanoparticles, hybrid microparticles, zeta-potential, antimicrobial activity

## Abstract

Silver nanoparticles (Ag NPs) conjugated with amino-functionalized cellulose nanofibrils (NH_2_−CNFs) were in situ-prepared by reducing silver ions with free amino groups from NH_2_−CNFs. The spectroscopy and transmission electron microscopy measurements confirmed the presence of non-agglomerated nanometer-in-size Ag NPs within micrometer-large NH_2_−CNFs of high (20 wt.-%) content. Although the consumption of amino groups during the formation of Ag NPs lowers the ζ-potential and surface charge of prepared inorganic–organic hybrids (from +31.3 to +19.9 mV and from 2.4 to 1.0 mmol/g at pH 7, respectively), their values are sufficiently positive to ensure electrostatic interaction with negatively charged cell walls of pathogens in acidic and slightly (up to pH ~8.5) alkaline solutions. The antimicrobial activity of hybrid microparticles against various pathogens (*Escherichia coli*, *Pseudomonas aeruginosa*, *Staphylococcus aureus*, and *Candida albicans*) is comparable with pristine NH_2_−CNFs. However, a long-timescale use of hybrids ensures the slow and controlled release of Ag^+^ ions to surrounding media (less than 1.0 wt.-% for one month).

## 1. Introduction

Nanocellulose (NC) is one of the most promising eco-friendly, non-toxic materials, obtained from renewable sources (plants, algae, tunicates, and some bacteria), with the potential to replace synthetic polymers in many applications due to their unique properties, including easy processability and the possibility to tailor morphology, specific surface area, crystallinity, and biocompatibility [1]. Because of these unique features, NC is recognized as a suitable material for various current and emerging applications, such as nanocomposites, adhesives, paints, barrier and functional coatings, fibers and filaments, films and foils, membranes and filters, biomedical and pharmaceutical products, supercapacitors, and batteries [1,2,3,4].

However, pristine NC does not display antimicrobial activity, and with the knowledge of the importance of non-toxic biodegradation for the environment, various approaches were proposed to obtain its antimicrobial activity. Generally, two different approach types are recognizable, the first based on surface modification of NC with aldehyde, amino, and quaternary ammonium groups [5], and the second based on conjugation with metal (Au, Ag, and Cu) [6,7,8], metal oxide nanoparticles (CuO, MgO, ZnO, and TiO_2_) [9,10,11,12,13], biocidal agents such as gentamicin [14,15,16], or antimicrobial polymers [4,17,18].

Functionalization of NC with amino groups is of particular interest. On one side, amino-functionalized cellulose nanofibrils (NH_2_−CNFs) display antimicrobial activity due to the electrostatic interaction between positively charged amino groups and negatively charged cell walls that enhanced their contact with microorganisms [19,20,21]. On the other side, the free amino groups are capable of reducing Ag^+^ ions to their metallic form, providing the possibility to prepare free-standing colloidal Ag NPs [22,23] or Ag NPs linked to different types of supports, polymer [24,25,26] and inorganic [27,28], with high antimicrobial activity. Besides the antimicrobial activity of Ag NPs on support, when they are easily accessible to microbial species in the surrounding medium, Ag NPs dispersed within a polymer matrix also have antimicrobial activity. Recently, we demonstrated that thin films prepared from CNF loaded with less than 0.5 wt.% of nanometer-in-size Ag NPs inhibited the growth of *E. coli* after five repeated cycles, indicating that they might serve as a sustainable replacement for their synthetic counterparts in different applications, from food packaging as a thin film [29] or functional coating [30], to textiles [31], up to the medical/pharmaceutical [27,28] industry.

This study thus aims to evaluate the antimicrobial activity of a hybrid based on amino-functionalized cellulose nanofibrils (NH_2_−CNFs) decorated with Ag NPs, prepared in situ, taking advantage of the reducing ability of free amino groups. So, combining inorganic (Ag NPs) and organic (NH_2_−CNFs) components into a hybrid (Ag/NH_2_−CNFs), with the knowledge that each of the components displays antimicrobial activity [29,30,31,32], was intended to improve the antimicrobial performance of the hybrid under long-term working conditions. Samples were characterized in detail before determination of their minimum inhibitory concentrations (MICs) and minimum bactericidal concentrations (MBCs) against different microbial species (Gram-negative bacteria *Escherichia coli* and *Pseudomonas aeruginosa*, Gram-positive bacterium *Staphylococcus aureus*, and yeast-type fungi *Candida albicans*). In addition, the content of the inorganic phase was determined by thermogravimetric measurements, the optical property of hybrids originating from Ag NPs was correlated with transmission electron microscopy data, and X-ray diffraction analysis provided the influence of the inorganic component on the crystallinity of NH_2_−CNFs. Finally, as a crucial factor influencing the electrostatic interaction of samples with the bacteria’s wall, the pH-dependent total charge and ζ-potentials of samples in different media were determined, including the hydrodynamic size of the hybrids.

## 2. Experimental

### 2.1. Materials

Wood-based chain-like cellulose nanofibrils (CNFs) with diameters in the 10–70 nm range and length of a few micrometers (1–3 µm) were prepared from bleached softwood pulp, refined in a multistage disc refiner, and supplied by the University of Maine (Orono, ME, USA), The Process Development Center, in the USA. The thorough microscopic characterization of CNFs can be found in our previous studies [31,32], but for clarity, the SEM image of CNFs is provided in the Supplementary Materials (Figure S1). Hexamethylenediamine (HMDA, 98% purity) and all other chemicals used were purchased from Sigma-Aldrich Co. Ltd. (St. Louis, MO, USA) and used without further purification.

### 2.2. Preparation of Amino-Functionalized CNFs (NH_2_−CNFs) Decorated with Silver Nanoparticles (Ag NPs)

**Scheme 1:**
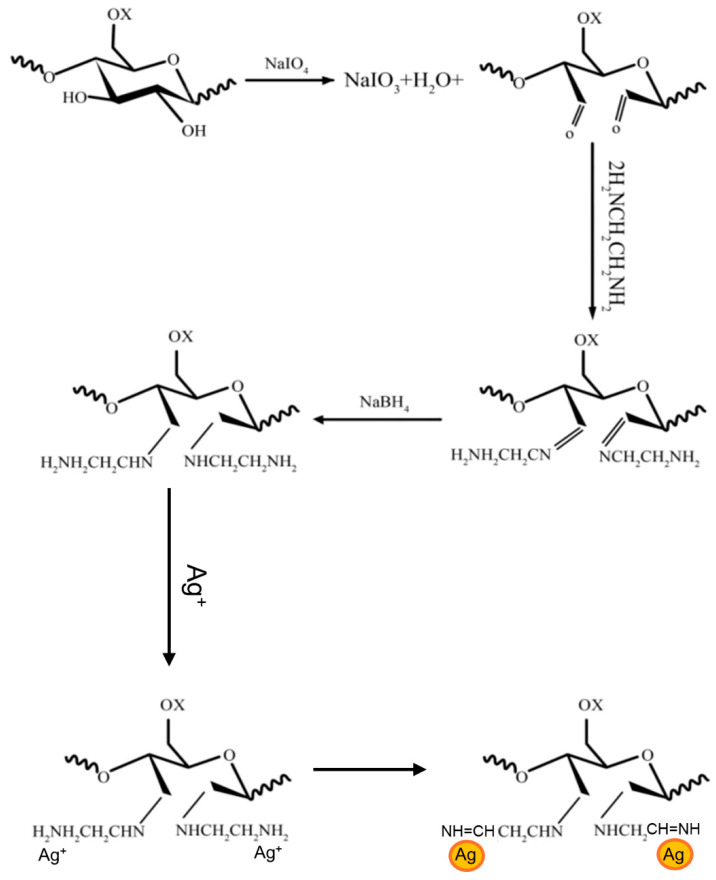
Synthetic pathway for preparation of inorganic–organic hybrid consisting of Ag NPs and amino-functionalized CNFs.

First, the amino-functionalized CNFs (NH_2_−CNFs) were prepared by a two-step reaction, as presented in Scheme 1 following our previous study [32]. Briefly, the aqueous dispersion of CNFs (1 wt.%, 100 mL) was first oxidized to exhibit aldehyde functional groups at the C2 and C3 cellulose unit positions by mixing it with pre-dissolved sodium periodate (1.6 g NaIO_4_ per 1 g of CNF) and stirring for 48 h in the dark at room temperature. The product was washed thoroughly a few times with deionized water for up to 3 days. The functionalization of pre-oxidized CNFs with HMDA was performed through a Schiff base reaction via aldehyde groups. For that purpose, 200 mL of 0.5 wt.% pre-oxidized CNF suspension was ultrasonicated for 5 min, and then 8.0 mmol of HMDA was added. The mixture was stirred continuously for 6 h at 30 °C, followed by in situ reduction of the resulting imine intermediate at room temperature, employing 0.58 g of NaBH_4_. The products (pre-oxidized CNFs and NH_2_−CNFs) were rinsed several times with deionized water, monitoring the washing procedure spectrophotometrically.

In the next step, the Ag NPs were grown onto NH_2_−CNFs by the in-situ reduction of Ag^+^ ions, taking advantage of the reducing ability of free amino groups, which are present in the amino-functionalized CNFs [26]. Briefly, the mixture with a 1:1 molar ratio between Ag^+^ ions and amino groups (85 mg of AgNO_3_ and 2.1 mL of amino-functionalized CNFs in 50 mL of H_2_O) was stirred overnight under reflux at 60 °C. This ratio was chosen to ensure that all added Ag^+^ ions would be reduced, leaving no ionic silver in the solution. The appearance of a yellow-brown color indicated the successful reduction of Ag^+^ ions to metallic silver. Then, the solid product was separated from the solution by centrifugation (10 min at 10,000 rpm), washed several times with deionized water, and dried at 40 °C in a vacuum oven for 24 h. For clarity reasons, Ag NPs conjugated with NH_2_−CNFs will be denoted further in the text as Ag/NH_2_−CNFs.

### 2.3. Characterization of NH_2_−CNFs and Ag/NH_2_−CNFs

Absorption of NH_2_−CNFs and Ag/NH_2_−CNFs was evaluated in the UV–Vis–NIR spectral range by diffuse reflectance measurements (Shimadzu UV–Visible UV-2600 spectrophotometer equipped with an integrated sphere ISR-2600 Plus, Shimadzu Corporation, Kyoto, Japan).

The content of metallic silver in Ag/NH_2_−CNFs was determined by thermogravimetric analysis (TGA) using a Setaram Setsys Evolution-1750 instrument. In order to completely degrade the organic part (CNF), the TGA measurements were thus performed under a dynamic air atmosphere (flow rate of 20 cm^3^/min) in the temperature range from room temperature to 1000 °C (heating rate of 10 °C/min). The content of released silver (Ag^+^ ions and Ag NPs) after 30 days of Ag/NH_2_−CNF incubation in distilled water at room temperature was determined using inductively coupled plasma–optical emission spectroscopy (ICP-OES Thermo Scientific iCAP 7400, Waltham, MA, USA).

X-ray diffraction (XRD) measurements of the prepared samples were carried out using a Rigaku SmartLab instrument (Tokyo, Japan) with Cu Kα1,2 radiation. The measurements were performed with continuous scanning at 2°/min and by collecting the data at 0.02° intervals.

Microstructural characterization of the Ag/NH_2_−CNFs was performed on a transmission electron microscope (TEM) JEOL JEM-2100 LaB6 (Tokyo, Japan) operating at 200 kV. TEM images were acquired with a Gatan Orius CCD camera (Pleasanton, CA, USA) at 2× binning.

Potentiometric titrations of native and modified CNF samples suspended in milli-Q water were performed to quantify the process-dependent surface charge contributions. The titration was carried out using a dual-burette instrument (Mettler Toledo T-70, Toledo, Ohio, USA) equipped with a combined glass electrode (Mettler TDG 117, Toledo, Ohio, USA) and filled with 0.1 M HCl (Merck, Titrisol, Darmstadt, Germany) and 0.1 M KOH (Baker, Dilut-it, Radnor, PA, USA). Samples soaked in milli-Q water were rinsed in a low-pH solution (0.01 M HCl) to convert the basic and acidic groups into protonated forms and then dried at 40 °C. The titrations were carried out at room temperature (23 ± 1 °C), forward and backward between pH 2 and 11. The molar concentration, related to the overall charge of the side groups, was calculated from the potentiometric titration data. All the reported values are the mean values of duplicate determinations.

The hydrodynamic size and zeta-potential of the native and modified CNF samples, suspended in different media (water and mixture of water–ethanol/acetone with various contents of organic solvents) were assessed by dynamic light scattering (DLS) using a Zetasizer, Nano ZS ZEN360 (Malvern Instruments Ltd., Malvern, UK), at 25 ± 0.1 °C, and the DTS1070 disposable folded capillary cell. The analysis was performed by applying the following parameters: refractive index of cellulose (1.47), refractive indexes of solvents (1.33, 1.363, and 1.357, for water, ethanol, and acetone, respectively), and viscosities (0.8872, 1.1734, and 0.3084 cP for water, ethanol, and acetone, respectively). A field of 150 V was applied across the nominal electrode spacing of 16 mm. The samples were prepared at concentrations of 0.01 wt.-% and measured at around pH 7 after dispersing them at 10,000 rpm for 3 min using Ultraturax IKA GmbH (Staufen im Breisgau, Germany). The presented values are the averages from at least two individual measurements.

### 2.4. Evaluation of the Antimicrobial Ability of NH_2_−CNFs and Ag/NH_2_−CNFs

The minimum inhibitory concentrations (MICs) and minimum bactericidal concentrations (MBCs) of the NH_2_−CNF and Ag/NH_2_−CNF suspensions were provided by the National Laboratory of Health, Environment, and Food (NLZOH), Maribor, Slovenia, according to the standard test method E2149-10 using Gram-negative bacteria *Escherichia coli* (*E. coli*, DSM 1576) and *Pseudomonas aeruginosa* (*P. aeruginosa*, DSM 1128), Gram-positive bacterium *Staphylococcus aureus* (*S. aureus,* DSM 799), and yeast-type fungi *Candida albicans* (*C albicans*, DSM 1386) as testing microorganisms. This test method evaluates the antimicrobial performance of immobilized antimicrobial materials.

Briefly, 0.1 mL bacteria cell suspensions, in the concentration range of 1.0 × 10^7^–1.0 × 10^8^ Colony-Forming Units (CFU/mL), and fungal cell suspension of 1.5 × 10^5^ CFU/mL were prepared and added to the NH_2_-CNF and Ag/NH_2_-CNF suspensions that were serially diluted using 15.5 and 24.0 mg/mL stock solutions, respectively. Initial concentrations of microbial species were determined by comparing the turbidity of microbial suspensions with that of the McFarland standards. After the 24 h contact, microorganism growth was followed by tube opacity, and dilution when the microbial growth was not detectable was considered the MIC value. The control experiments, i.e., experiments without any sample in the microorganism culture, were performed similarly.

The MBC values were evaluated on Mueller–Hinton plates by sampling tubes without noticeable microbial growth, excluding NH_2_-CNF and Ag/NH_2_-CNF. The MBC values are the lowest NH_2_-CNF and Ag/NH_2_-CNF concentrations when microbial growth on the solid medium does not occur.

## 3. Results and Discussion

The synthesis of inorganic–organic hybrid particles consisting of Ag NPs and amino-functionalized cellulose nanofibrils (NH_2_−CNFs) is a two-step process (Scheme 1). In the first step, NH_2_−CNFs were prepared using a straight-forward procedure based on CNF oxidation by sodium periodate (NaIO_4_) to obtain dialdehyde nanocellulose followed by a reaction with ethylenediamine (C_2_H_4_(NH_2_)_2_) through an imine intermediate, sodium borohydride (NaBH_4_), that provided the final product, NH_2_−CNFs [31,33].

The high content of free amino groups, estimated to be around 5.0 mM/g, is a significant feature of NH_2_−CNFs. The amino groups are reactive and capable of reducing silver ions to metallic silver, free [22,23], or attached to inorganic [27,28] or organic [24,25,26] supports. So, in the second synthetic step (Scheme 1), Ag NPs were in situ prepared by the electron transfer reaction from the amino groups and their transformation into imino groups, and thus conjugated to CNFs over the lone electron pairs of the N atoms from amino groups [23,27,28]. The abundant presence of free amino groups in NH_2_−CNFs (Table 1) provides the possibility of preparing micro-large, inorganic hybrid micro-particles with a high content in the inorganic phase. Accordingly, the equimolar ratio between free amino groups and Ag^+^ ions was used to prepare the Ag/NH_2_−CNFs hybrid particles with potential application for wastewater disinfection.

**Figure 1:**
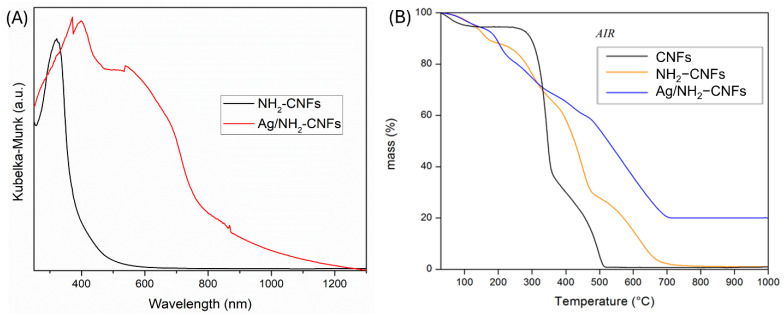
(**A**) Kubelka-Munk transformations of reflection data of NH_2_−CNFs and Ag/NH_2_−CNFs. (**B**) TG curves, measured in the air at a heating rate of 10 °C/min, of CNFs, NH_2_−CNFs, and Ag/NH_2_−CNFs.

Before antimicrobial tests, the samples obtained in each synthetic step were thoroughly characterized. Kubelka-Munk transformations of reflection data of NH_2_−CNFs and Ag/NH_2_−CNFs films were performed to follow Ag NPs formation, identified by the optical changes, as shown in Figure 1A. Opposite to NH_2_−CNFs, the Ag/NH_2_−CNFs hybrid absorbs in the visible and near-infrared spectral region with surface plasmon resonance bands peaking around 400 and 550 nm, indicating a relatively small size of slightly agglomerated metallic silver. The peak at around 400 nm is not well-resolved due to overlap with the absorption of the NH_2_−CNFs.

The TG measurement in the air was applied to evaluate the content of the inorganic phase in the hybrid (Figure 1B). The residual mass of CNFs and NH_2_−CNFs samples at high temperatures (>700 °C) was negligible. On the other hand, the residual mass of the Ag/NH_2_−CNFs, corresponding to the content of metallic silver, was around 20 wt.%. Based on the concentration of precursors, free amino groups in NH_2_−CNFs, and Ag^+^ ions, the calculated maximal content of metallic silver is 35 wt.%. The experimentally measured lower inorganic phase content is the most likely consequence of amino groups’ steric hindrance during the Ag NPs formation.

**Figure 2:**
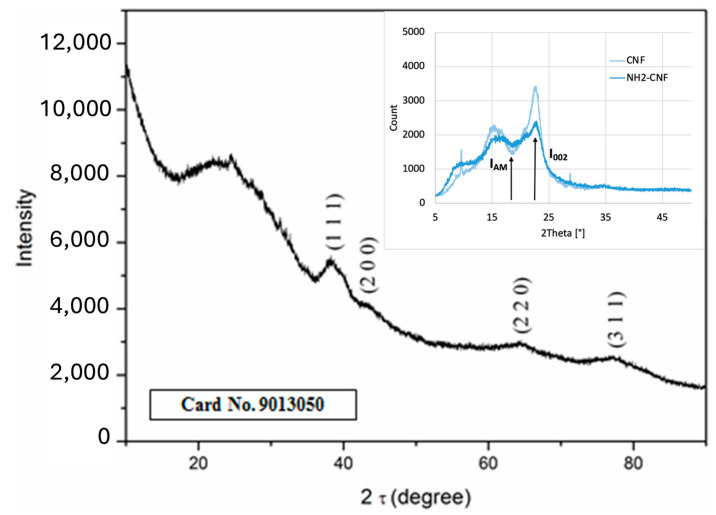
The XRD pattern of the Ag/NH_2_−CNFs hybrid; inset: the XRD patterns of CNFs and NH_2_−CNFs.

The wide-angle XRD pattern of the Ag/NH_2_−CNFs hybrid, presented in Figure 2, gave diffraction peaks at 38.1, 43.7, 64.3, and 77.3° belonging to the (111), (200), (220), and (311) crystal planes of face-centered-cubic silver (Card No: 9013050). The average crystallite size of silver was estimated to be around 5 nm based on the half-width of diffraction peaks and using Scherrer’s equation.

The XRD spectra of CNFs and NH_2_−CNFs, shown in the inset of Figure 2, correlated with the literature data [34], exhibiting the principal peaks around 16.5, 22.5, and 35° which is attributed to the overlapping (1–10) and (110), (200) and (004) planes of monoclinic cellulose, respectively. The empirical equation was applied to calculate the crystallinity index (CI) of CNFs and NH_2_−CNFs using the intensity of the highest XRD peak (I_002_) and the minimum intensity at a position between the (002) and the (101) peaks (I_AM_), which was at about 18.3° [35]:

(1) $$\mathrm{CI}=\frac{I_{002}-I_{\mathrm{AM}}}{I_{002}}\times 100\%$$

The crystallinity indexes of CNFs and NH_2_−CNFs were found to be around 58.3 and 32.7%, indicating that the functionalization steps (above all, the oxidation phase) slightly influenced the crystal-like arrangement of hydrogen-bonded CNFs and consequently their morphology [36].

**Figure 3:**
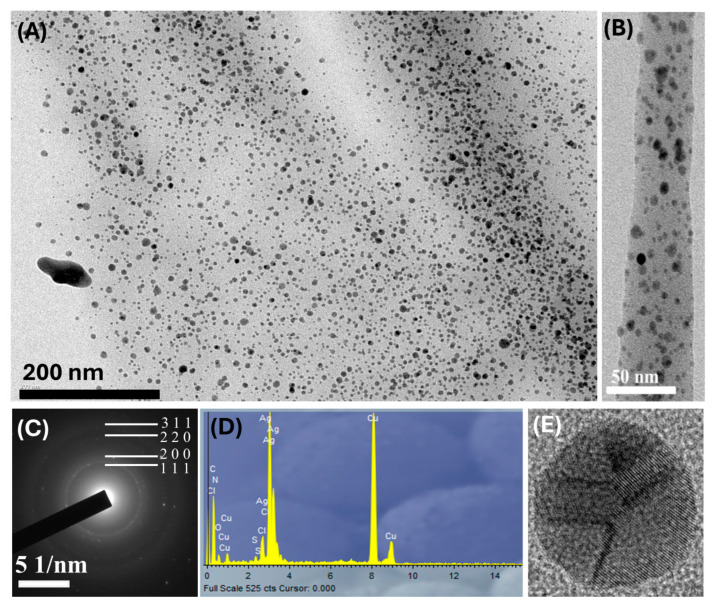
Low-magnification TEM images of Ag/NH_2_−CNFs composite (**A**,**B**) and corresponding SAED pattern (**C**) as well as EDX spectrum (**D**). High-magnification TEM image of typical Ag NP attached to NH_2_−CNFs support (**E**).

The thorough morphological characterization of the Ag/NH_2_−CNFs composite was performed using TEM. Low-magnification TEM images of Ag/NH_2_−CNFs composite (Figure 3A,B) indicated the presence of randomly distributed spherical nanometer-sized (<10 nm) Ag NPs across the surface of the NH_2_−CNFs (Figure 3B), although agglomeration is seldom noticeable. So, there was a good agreement concerning the size estimation of Ag NPs between TEM, XRD, and spectroscopy data. Analysis of the selected area electron diffraction (SAED) pattern (Figure 3C) revealed the presence of diffraction rings consistent with the inverse face-centered-cubic crystalline silver structure, and the analysis of the EDX spectrum (Figure 3D) showed the presence of a pronounced peak corresponding to silver. Finally, a high-resolution TEM image displays typical Ag NPs attached to NH_2_−CNFs support (Figure 3E). The morphological changes occurring in each step of the amino-functionalization process of CNFs, including the influence of dispersion media, were in detail described in our recent publications [31,32] and are omitted in the present study.

**Figure 4:**
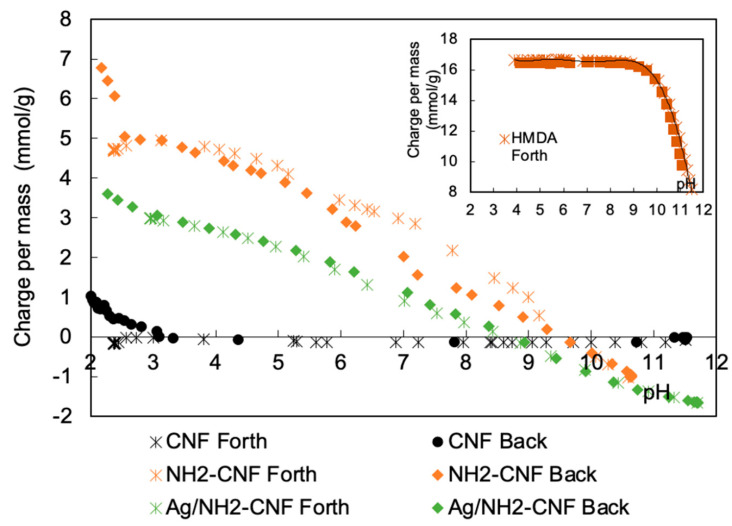
Potentiometric titration curves of native CNFs, NH_2_-CNF, and Ag/NH_2_-CNF with corresponding charges suspended in water; concentration: 0.001 wt.%. Inset: the titration curves (forth and back) of pure HMDA.

**Figure 5:**
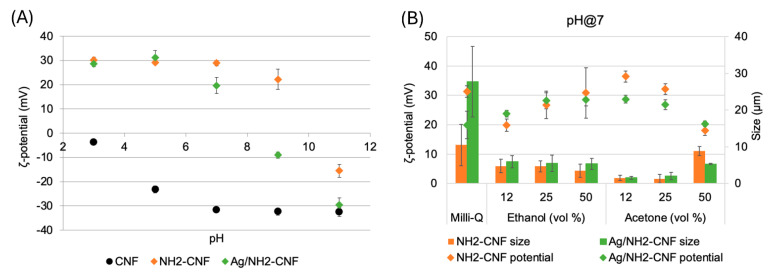
(**A**) ζ-potentials and (**B**) average hydrodynamic size distribution of native CNFs, NH_2_-CNF, and Ag/NH_2_-CNF in water and water containing different volume percent of ethanol or acetone at pH 7, analyzed by DLS; concentration: 0.001 wt.%.

It is well-known that the surface charge of Gram-positive and Gram-negative bacteria is negative in media of different acidities and ionic strengths [37]. Representative of Gram-negative bacteria, *E. coli*, has a more negatively charged cell wall than typical Gram-positive bacteria, *S. aureus*. So, it is crucial to determine the pH-dependent surface charge and ζ-potential of all samples (CNFs, NH_2_−CNFs, and Ag/NH_2_−CNFs) from the electrostatic interaction point of view. Also, the potentiometric titration curves (Figure 4) and the pH-dependent ζ-potential values (Figure 5A) indicated chemical changes occurring during the synthetic route from CNFs over NH_2_−CNFs to Ag/NH_2_−CNFs. The native CNFs are negatively charged in the entire pH region, showing a negligible bend of negative charge (0.12 mmol/g) at pH around 4–5, related to the seldom presence of anionic surface groups, preferably carboxylic, formed during the preparation of CNFs or due to residual lignin, responsible for ζ-potential of around -31.6 mV at pH 7 (see Table 1).

The titration curves (forth and back) of pure HMDA (inset in Figure 4) show a steady high positive charge in a broad pH range, up to the pH 10, close to its deprotonation constant (pK = 11; [38]) where a steep decrease takes place. So, the functionalization of CNFs with HMDA, followed by the introduction of amino groups, leads to positively surface-charged NH_2_−CNFs (Figure 4) at pH values lower than 9. Also, the titration curves of the NH_2_−CNFs have different shapes compared to the native HMDA. The gradual increase in positive charge is noticeable by increasing the acidity with a barely noticeable bend in the intermediate pH range, confirming the consumption of one of the amino groups from HMDA during the functionalization of CNFs and yielding a total charge of around 5.99 mmol/g. In particular, at pH 7, where the material property is the most important from the applicative side, the ζ-potentials of the NH_2_−CNFs is +31.3 mV with 2.44 mmol/g of functional groups. These results agree with our recently published data [31,32].

The formation of Ag NPs and their conjugation to functionalized CNFs over amino groups follows a significant decrease in surface charge and ζ-potential (Figure 4 and Figure 5A, respectively). Consequently, the total charge is about 4.64 mmol/g, and at pH 7, ζ-potential is +19.9 mV with corresponding 1.0 mmol/g functional groups (Table 1). The presence of remaining free amino groups in Ag/NH_2_−CNFs indicates non-stoichiometrical conjugation of Ag NP to NH_2_-CNF, and it can be related to the formation mechanism of metallic silver rather than individual conjugation. The pH-dependent potentiometric titration and ζ-potential measurements are in agreement with the thermogravimetric estimation of the content of silver in Ag/NH_2_−CNFs, found to be lower than expected for the stoichiometric reaction between Ag^+^ ions and amino groups.

In addition, the ζ-potentials and the average size of NH_2_−CNFs and Ag/NH_2_−CNFs were thoroughly analyzed in mixed solvents, water-ethanol or water-acetone of different compositions, at pH 7, and compared with results obtained in water (Figure 5B). It was confirmed that the presence of hydrophobic amino-bearing molecules (HMDA contains ethyl units) attached to CNFs induced their aggregation in water, which can be reduced in the presence of highly polar solvents [31,32]. First, the hydrodynamic size analysis of the Ag/NH_2_−CNFs dispersed in water revealed the presence of significantly larger micro-sized particulates (27.7 μm) compared to the NH_2_−CNFs with high polydispersity and average size of 10.5 μm. Generally, the formation of several micrometers in size NH_2_−CNFs is related to a both-sides (crosslinking) attachment of HMDA to pre-oxidized CNFs that also occurred, besides one-side (grafting) reaction, as well as the formation of aggregates due presence of hydrophobic methylene chain in attached HMDA, as already established in our previous studies [31,32]. On the other hand, a diminished ζ-potential upon the attachment of Ag NPs to the NH_2_−CNFs support, from +31.3 to +19.9 mV (Table 1 and Figure 5B), i.e., the decrease in surface charge (Figure 4) is responsible for the formation of significantly larger Ag/NH_2_−CNFs hybrids. However, the differences in the ζ-potentials between NH_2_−CNFs and Ag/NH_2_−CNFs are almost negligible for any composition of studied mixed solvents. For example, ζ-potentials of 4.7–3.5 μm large NH_2_−CNFs in milli-Q water containing 12, 25, and 50 vol.% of ethanol are about +20.0, +27.0, and +31.5 mV, respectively, while corresponding values for the 5.9–5.3 μm large Ag/NH_2_−CNFs are +23.8, +28.1, and +28.5 mV, respectively. As a consequence of the similar ζ-potentials in mixed solvents, the hydrodynamic sizes of NH_2_−CNFs and Ag/NH_2_−CNFs, determined by the DLS, are close to each other, within experimental errors, significantly smaller compared to being dispersed in water, and in all cases around 5 μm. Opposite to the mixed water–ethanol solvent, the increase in the volume percent of aprotic acetone (does not contain an O−H group available for H bonding) in water leads to a decrease in ζ-potentials from around +36 to +19 mV for both NH_2_−CNFs and Ag/NH_2_−CNFs. The hydrodynamic size of NH_2_−CNFs and Ag/NH_2_−CNFs is less than 5 μm, except for the mixture with the highest content of acetone (50 vol.%), where both samples have the lowest ζ-potentials (around +20 mV).

To evaluate the antimicrobial ability of the prepared Ag/NH_2_−CNFs, minimum inhibition concentrations (MICs) and minimum bactericidal concentrations (MBCs) were measured against Gram-positive bacteria *S. aureus*, Gram-negative bacteria *E. coli* and *P. aeruginosa*, and yeast-type fungi *C. albicans*, and compared with the antimicrobial ability of NH_2_−CNFs. This dilution method provides concentration-dependent antimicrobial activity measurements of prepared samples diluted 2 times up to 128 times. The concentrations of NH_2_−CNF and Ag/NH_2_−CNF stock solutions were 15.5 and 24.0 mg/mL, respectively, and the content of silver in the Ag/NH_2_−CNFs was around 20 wt.%.

The CNFs have no inherent antimicrobial activity [32,39,40], so the control sample did not show any antimicrobial activity. As expected, the NH_2_−CNFs display growth inhibition of all studied microbial species. The MIC values obtained in this study and published MIC values from our previous studies [31,32] are presented in Table 2. Besides the hydrophobic tail, the presence of the protonated, positively charged amino groups is essential for the efficient antimicrobial activity of NH_2_−CNFs since both the hydrophobic and electrostatic interactions with the hydrophobic and negatively charged cell walls lead to increased contact with microorganisms [41], compromising their integrity and leading to the leakage of cytoplasmic content and, ultimately, cell lysis, thereby resulting in a bactericidal effect [20]. The differences in the MIC values of NH_2_−CNFs, particularly for Gram-positive bacteria *S. aureus* and Gram-negative bacteria *E. coli*, are the most likely consequence of the different testing methodologies causing differences in the dispersibility of samples.

It is well known that S. aureus has a better defense system against Ag^+^ ions than *E. coli.* [42]. However, *E. coli* has a more negatively charged wall than *S. aureus* [37]. The stronger electrostatic interaction with slowly released Ag^+^ ions might be the reason for the altered resistance of bacteria, i.e., the lower MIC values of Gram-negative bacteria *E. coli* and *P. aeruginosa* compared to Gram-positive bacteria *S. aureus* (Table 2). Also, the same MIC values for Gram-positive bacteria (*E. coli, P. aeruginosa*) and yeast-type fungi *C. albicans* are most likely the consequence of the slow release of Ag^+^ ions.

Also, besides MIC, the MBC values for NH_2_−CNFs and Ag/NH_2_−CNFs are also collected in Table 2, and based on these data, we can conclude the following: First, both composites display growth inhibition of all studied microbial species. On the other hand, the microorganism-killing activity that prevents their colonization occurs either at higher silver concentrations than inhibition or does not take place in the case of *S. aureus* in the studied concentration range. Second, the MIC values are higher than the ones obtained for free-standing similar-in-size Ag NPs [41] or immobilized Ag NPs on glass support [43]. However, MIC and MBC values depend on the morphology (size and shape), preparation method, and surface properties of Ag NPs. So, a similar MIC value against *S. aureus* (0.625 mg/mL) was reported by Parvekar et al. [44] for 5 nm-in-size Ag NPs, while Ayala-Núñez et al. [45] demonstrated that the MIC and MBC of 10 nm Ag NPs are in concentrations of 1.35 mg/mL against *S. aureus*. Third, the MIC values obtained for Ag/NH_2_−CNFs are lower than those for NH_2_−CNFs. Although free amino groups are present in the Ag/NH_2_−CNFs, most likely due to their steric hindrance, the mechanism of the antimicrobial action of the Ag/NH_2_−CNFs is different, switching to the antimicrobial activity of Ag NPs.

The antimicrobial action of immobilized Ag NPs can be due to direct contact of microbial species with Ag NPs, immobilized or detached in solution, and with released Ag^+^ ions in solution [46]. The advantages of immobilized over colloidal Ag NPs are, as shown by Lv et al. [47], their higher stability and consequently suppressed oxidation that provides long-term antimicrobial activity. From the practical point of view, low and controlled release of Ag NPs and Ag^+^ ions into wastewater is the essential prerequisite for its disinfection. So, we aged the Ag/NH_2_−CNFs composite in water and, after one month, applied the ICP-AES technique to determine the concentration of silver in the supernatant. The ICP-AES measurement revealed that the total concentration of released silver is less than 1% of the content of Ag NPs in the Ag/NH_2_−CNF composite. The ICP-AES technique does not distinguish the valence state of silver, and since the supernatant was yellowish, the presence of detached Ag NP from the composite in the supernatant was proven by spectroscopy measurements. The absorption spectrum of the supernatant (Supplementary Materials, Figure S2) has the surface plasmon resonance band peaking at 410 nm, characteristic of nanometer-in-size Ag particles. Nevertheless, although the Ag/NH_2_-CNF composite serves as a reservoir of silver, the antimicrobial activity will be present even after the complete release of silver since the NH_2_−CNFs can capture and deactivate the bacteria from wastewater [48].

It is well known that released silver in any form (ionic or metallic) retains its cytotoxicity and ecotoxicity even at a concentration as low as 1.0 mg/L [41,49]. So, a trade-off between the release rate and efficiency of the antimicrobial action is a prerequisite for the Ag NPs’ application as a disinfection agent. To conclude, considering the high content of Ag NPs in the Ag/NH_2_−CNFs (20 wt.-%), slow release of Ag^+^ ions to surrounding media, and negligible detachment of Ag NPs, it seems that the prepared sample is suitable for long-term antimicrobial action and worthy of further investigation of its potential application.

## 4. Conclusions

The free amino groups in the NH_2_−CNFs provided a simple way to prepare inorganic–organic hybrid particles with a high content of Ag NPs (20 wt.-%). Based on the characterization and antimicrobial activities of the prepared samples, several conclusions can be drawn.

- Incorporating nanometer-large Ag NPs does not significantly affect the hydrodynamic size, ζ-potential, or surface charge of NH_2_−CNFs, i.e., the hybrid retained desirable properties of its organic component.
- The inorganic–organic (Ag/NH_2_−CNFs) hybrid and NH_2_−CNFs display similar antimicrobial activity, although the mechanism of their antimicrobial action is different, i.e., electrostatic interactions via amino groups versus Ag^+^ ions released from Ag NPs.
- The slow release of Ag^+^ ions and negligible detachment of Ag NPs to surrounding media, i.e., the stability of the prepared hybrid, ensure its long-term antimicrobial action.

Although the antimicrobial activity of the inorganic–organic Ag/NH_2_−CNFs hybrid was tested on a limited number of pathogens, the selected ones (*Escherichia coli, Pseudomonas aeruginosa, Staphylococcus aureus,* and *Candida albicans*) are the most frequently used representatives of Gram-negative and Gram-positive bacteria, as well as yeast-type fungi. Based on the results, we believe that this study is a good starting point for further investigations toward potential applications of Ag/NH_2_-CNF hybrids with a high content of Ag NPs for wastewater treatment on a large scale.

## Figures and Tables

**Table 1:** The hydrodynamic size, ζ-potential, surface charge at pH 7, and a total charge of native CNFs, NH_2_−CNFs, and Ag/NH_2_−CNFs suspended in milli-Q water.

Sample	Size at pH 7 (μm)	ζ-Potential at pH 7 (mV)	Charge at pH 7 (mmol/g)	Total Charge (mmol/g)
CNFs	4.2 ± 1.0	−31.6 ± 1.0	0.14 ± 0.01	0.12 ± 0.01
NH_2_−CNFs	10.5 ± 5.6	+31.3 ± 1.9	2.44 ± 0.04	5.99 ± 0.04
Ag/NH_2_−CNFs	27.7 ± 9.6	+19.9 ± 4.7	1.00 ± 0.01	4.64 ± 0.04

**Table 2:** Minimum inhibition concentrations (MICs) and minimum bactericidal concentrations (MBCs) of NH_2_−CNFs and Ag/NH_2_−CNFs.

	NH_2_−CNFs				Ag/NH_2_−CNFs	
	MIC (mg/mL)			MBC (mg/mL)	MIC (mg/mL)	MBC (mg/mL)
*S. aureus*	0.96	~3 *	0.12 **	4 *	1.9	>12
*E. coli*	0.12	8 *	1.9 **	16 *	3.0	12
*P. aeruginosa*	0.48	n.d.	n.d.	n.d.	3.0	~12 ***
*C. albicans*	0.24	n.d.	0.24 **	n.d.	3.0	~12 ***

* Data from reference [33]. ** Data from reference [32]. n.d.—not determined. *** Minimal growth after 24 h.

## Data Availability

The authors declare that all the data generated or analyzed during this study are available within the article.

## Supplementary Materials

The following supporting information can be downloaded at: https://www.mdpi.com/article/10.3390/jfb15100304/s1, Figure S1: The SEM image of CNFs. Figure S2: Absorption spectrum of supernatant after 30 days in contact with Ag/NH_2_−CNFs.
