# Supplementary material for: Antimicrobial Activity of Amino-Modified Cellulose Nanofibrils Decorated with Silver Nanoparticles
Source: J Funct Biomater. 2024 Oct 13;15(10):304. doi: 10.3390/jfb15100304 (PMC11508708; doi:10.3390/jfb15100304)
Supplement: Supplementary file 1 [file jfb-15-00304-s001.zip › jfb-3240048-supplementary file.pdf]

Supporting Information

Antimicrobial activity of amino-modified cellulose nanofibrils decorated with silver nanoparticles

Vesna Lazić[1], Jovan M. Nedeljković[1*], Vanja Kokol[2*]

[1]Vinča Institute of Nuclear Sciences − National Institute of the Republic of Serbia, University of Belgrade, Centre of Excellence for Photoconversion, Belgrade, Serbia.
[2]Univeristy of Maribor, Faculty of Mechanical Engineering, Maribor, Slovenia.

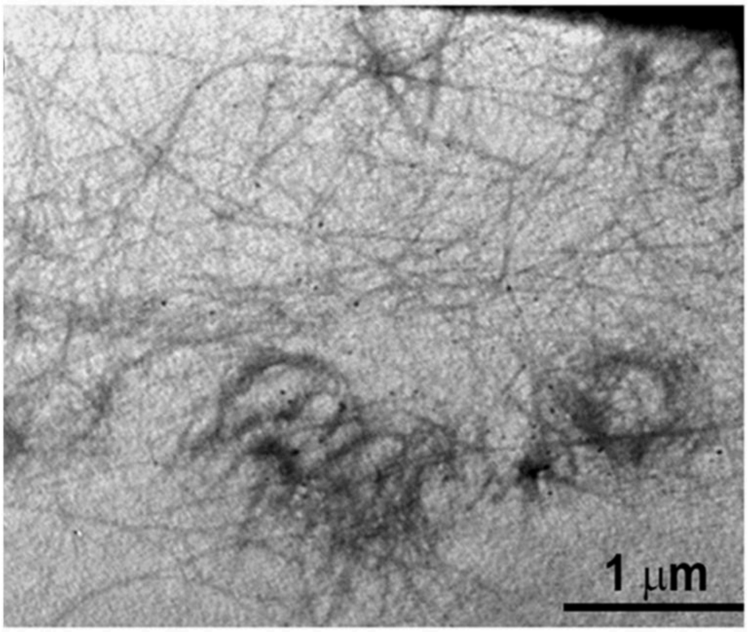

Figure S1. The SEM image of CNFs.

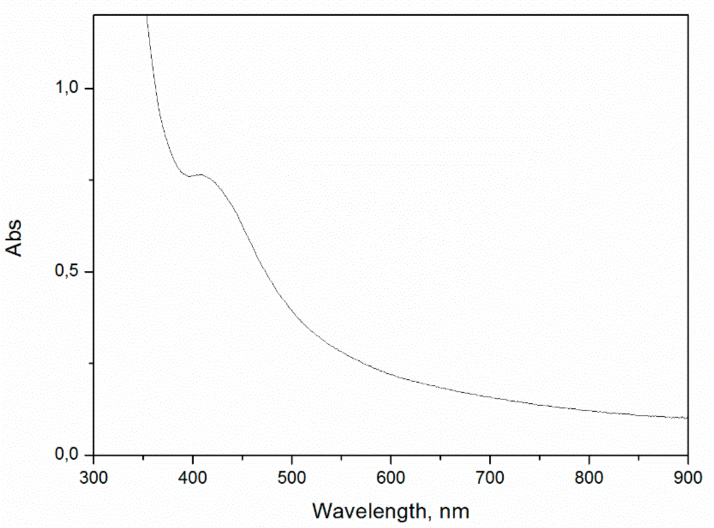

Figure S2. Absorption spectrum of supernatant after 30 days in contact with Ag/NH_2–CNFs.
